# Exploratory study of how Cognitive Multisensory Rehabilitation restores parietal operculum connectivity and improves upper limb movements in chronic stroke

**DOI:** 10.1038/s41598-020-77272-y

**Published:** 2020-11-20

**Authors:** A. Van de Winckel, D. De Patre, M. Rigoni, M. Fiecas, T. J. Hendrickson, M. Larson, B. D. Jagadeesan, B. A. Mueller, W. Elvendahl, C. Streib, F. Ikramuddin, K. O. Lim

**Affiliations:** 1 Division of Physical Therapy, Division of Rehabilitation Science, Department of Rehabilitation Medicine, Medical School, University of Minnesota, Minneapolis, USA; 2 Centro Studi Di Riabilitazione Neurocognitiva - Villa Miari (Study Center for Cognitive Multisensory Rehabilitation), Santorso, Vicenza Italy; 3 Division of Biostatistics, School of Public Health, University of Minnesota, Minneapolis, USA; 4 University of Minnesota Informatics Institute, Office of the Vice President for Research, University of Minnesota, Minneapolis, USA; 5 Division of Rehabilitation Science, Department of Rehabilitation Medicine, Medical School, University of Minnesota, Minneapolis, USA; 6 Department of Radiology, Medical School, University of Minnesota, Minneapolis, USA; 7 Department of Psychiatry, Medical School, University of Minnesota, Minneapolis, USA; 8 Center of Magnetic Resonance Research (CMRR), University of Minnesota, Minneapolis, USA; 9 Department of Neurology, Medical School, University of Minnesota, Minneapolis, USA; 10 Division of Physical Medicine and Rehabilitation, Department of Rehabilitation Medicine, Medical School, University of Minnesota, Minneapolis, USA

**Keywords:** Stroke, Stroke, Brain, Rehabilitation

## Abstract

Cognitive Multisensory Rehabilitation (CMR) is a promising therapy for upper limb recovery in stroke, but the brain mechanisms are unknown. We previously demonstrated that the parietal operculum (parts OP1/OP4) is activated with CMR exercises. In this exploratory study, we assessed the baseline difference between OP1/OP4 functional connectivity (FC) at rest in stroke versus healthy adults to then explore whether CMR affects OP1/OP4 connectivity and sensorimotor recovery after stroke. We recruited 8 adults with chronic stroke and left hemiplegia/paresis and 22 healthy adults. Resting-state FC with the OP1/OP4 region-of-interest in the affected hemisphere was analysed before and after 6 weeks of CMR. We evaluated sensorimotor function and activities of daily life pre- and post-CMR, and at 1-year post-CMR. At baseline, we found decreased FC between the right OP1/OP4 and 34 areas distributed across all lobes in stroke versus healthy adults. After CMR, only four areas had decreased FC compared to healthy adults. Compared to baseline (pre-CMR), participants improved on motor function (MESUPES arm *p* = 0.02; MESUPES hand *p* = 0.03; MESUPES total score *p* = 0.006); on stereognosis (*p* = 0.03); and on the Frenchay Activities Index (*p* = 0.03) at post-CMR and at 1-year follow-up. These results suggest enhanced sensorimotor recovery post-stroke after CMR. Our results justify larger-scale studies.

## Introduction

Despite current neurorehabilitation for adults with stroke in the United States, about 70% of adults with stroke (+ / − 7 million Americans) still are unable to use their affected hand in daily life and live on with significant disability for many years^1–5^, calling for more effective therapies to restore motor function, especially for those with severe motor impairments^4^. Usually neurorehabilitation for adults with stroke in the clinic focuses on individualized, task-specific functional training that promotes intensity, repetition, and specificity of practice.

Recently, however, brain imaging and behavioural studies indicate that sensory impairments, and proprioceptive deficits in particular, impact motor function and motor recovery, and that providing proprioceptive training may promote motor recovery after stroke^6–11^. In fact, specific short-term robot-aided proprioceptive training has shown to improve proprioceptive acuity in people with stroke^11^ although the effectiveness of long-term proprioceptive training on sensorimotor function after stroke still remains to be seen.

Considering that proprioceptive training has shown to improve motor function by only focusing on a singular joint, we reasoned that a therapy, which addresses somatosensory and multisensory functions of the whole upper limb and trunk, might have a better impact on recovery of upper limb motor functions post-stroke.

**Figure 1:**
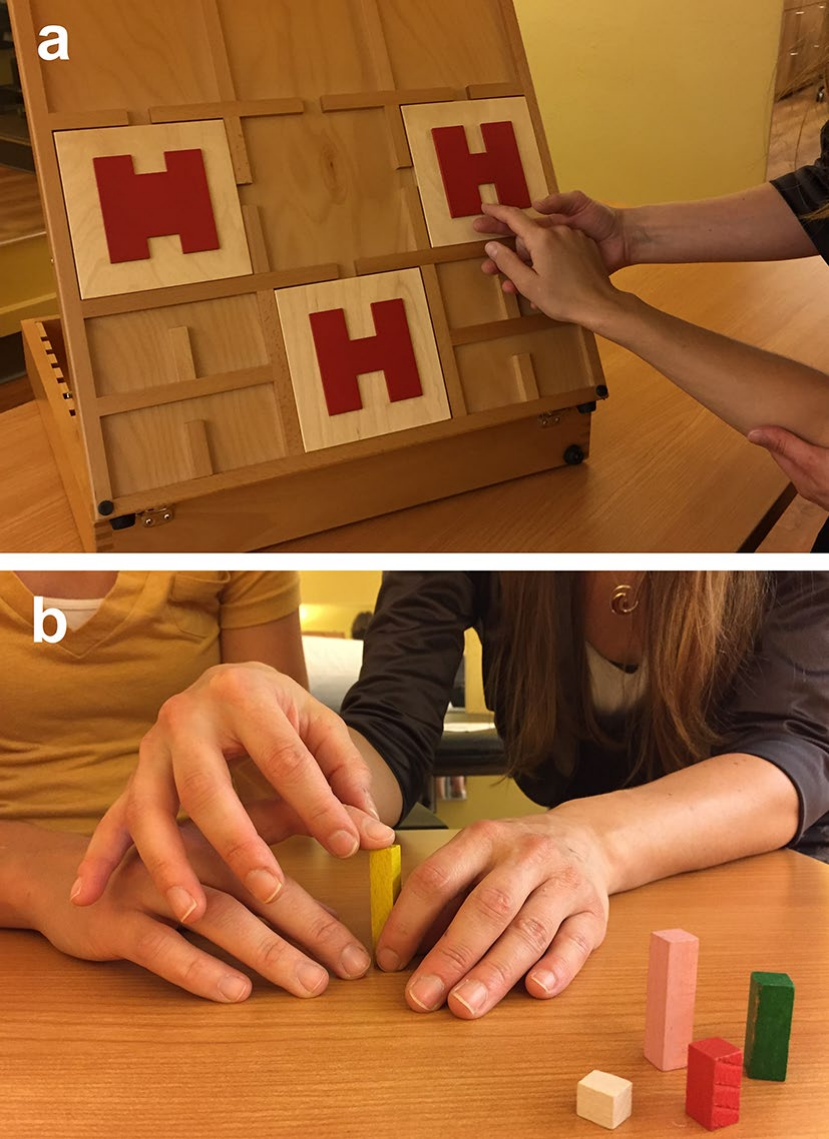
Shape discrimination exercise (**a**) and height discrimination exercise (**b**). The therapist first shows all the options to the participant. In (**a**) the therapist shows letters “H” with different widths of horizontal and vertical bars. Then the participant closes his or her eyes and the therapist guides the participant’s finger along the edged contour of one of the letters “H” to identify which letter “H” the therapist has chosen. The movement is felt in the shoulder joint. Through reflection and attention on associated shoulder movements, the patient feels the width and length of the horizontal and vertical bars and identifies the correct letter “H”. This kind of exercises provides an integrating of attention, sensory integration, reflection and awareness of body positions, movements, and feelings. Later in the session, this increased awareness of shoulder and arm movements will be evaluated during daily tasks that involve reaching. The latter will be done with eyes open. In (**b**) the wooden stabs have different heights. The participant first sees all the options. Then the participant closes his or her eyes and the therapist guides the participant’s finger along the edge of the wooden stab and stops at the top of the wooden stab. The participant has to feel the movement and position in the metacarpophalangeal joint of the finger while keeping the fingers relaxed, in order to identify the correct height of the wooden stab. Later in the session, this learned position and movement will be evaluated in a real-life situation in which, e.g., the participant integrates what was learned by opening the hand correctly to grasp a bottle.

Cognitive Multisensory Rehabilitation (CMR) indeed addresses somatosensory and multisensory functions of the whole body^12–14^. CMR is a sensorimotor rehabilitation approach, in which the patient is asked to solve sensory discrimination exercises with eyes closed or to solve multisensory discrimination exercises, e.g., by comparing feeling shapes with seeing shapes^12–14^ (Fig. 1). CMR is based on a therapist-guided approach in which cognitive processes are prompted by asking the patient to reflect on how the limb was moved or was positioned, and how the movement was felt in the body, and to pay attention to how the limb movements are related to other parts of the body and to spatial parameters in the environment^12–14^.

In short, CMR is focused on integrating body parts and on integrating the movements in relation to the environment during functional tasks. Two studies have shown that CMR could be effective for motor recovery for adults with acute stroke, especially for those with severe motor impairments, as well as in adults with chronic stroke^15,16^. Taken together, it is worth examining how CMR affects sensorimotor recovery of adults with stroke, particularly the underlying brain mechanism behind CMR.

To answer this question, we previously identified brain areas that were activated during one of the CMR exercises, i.e. shape discrimination, in healthy adults and adolescents, adults with stroke and children with cerebral palsy using our custom-build Magnetic Resonance Imaging (MRI)-compatible robot^17–20^. Based on our carefully controlled trials, we found that the cognitive processing of discriminating shapes—i.e. creating a mental picture of the first shape, keeping it in working memory to compare the distinct features of the first shape with the second shape—activated frontoparietal brain areas, including parts 1 and 4 of the parietal operculum (OP1/OP4, also known as the secondary somatosensory cortex, SII), insula, and crus I/II of the cerebellum^17–20^.

Interestingly, those crucial brain areas (i.e., OP1/OP4, insula and Crus I/II) were activated only when participants performed such cognitive processing or paid attention to the movement^17,20^. This discovery was significant because it revealed that OP1/OP4 might be preferentially activated when mindful attention is given to the movement.

As indicated by recent resting-state functional connectivity (FC) studies in healthy adults, OP1 and OP4 are an important part of the multimodal integration network in which visual, motor, and somatosensory information are integrated to form visuospatial body maps in the posterior parietal cortex (PPC)^21–24^. The visuospatial maps are then used to guide and control motor actions^21,22^. Therefore, it is possible that CMR could influence the multimodal integration network via OP1/OP4.

Not surprisingly, deficits in any step of the sensorimotor integration pathway contribute to motor function deficits post-stroke^25^. Indeed, adults with stroke who have motor impairments can have alterations in brain connectivity at all stages of this multimodal integration process, from primary sensory areas to associative multisensory regions (e.g., PPC)^26^.

All these observations led to the idea that CMR might elicit its effect on motor recovery post-stroke through improving connectivity within the multimodal integration network. More specifically, we hypothesized that CMR might restore resting-state FC between OP1/OP4 and other parts in the brain.

To explore this idea, we conducted a pilot study to see (i) if and how OP1/OP4 connectivity is reduced post-stroke compared to healthy adults; and (ii) if CMR restores the affected OP1/OP4 connectivity alongside recovery of sensorimotor function in adults with chronic stroke and upper limb motor impairments.

In this exploratory study, we reported on a limited sample size of 8 adults with stroke compared to 22 healthy adults for two main reasons: First, in order to obtain interpretable brain imaging data showing the effect of CMR on resting-state functional connectivity, we selected a homogeneous group of adults with stroke in terms of lesion location appearing in only one hemisphere, so that changes, if any, in the brain after CMR could easily be compared among these participants. Second, we selected right hemispheric stroke lesions rather than left hemispheric lesions in order to exclude patients with aphasia, which rarely occurs in adults with right hemispheric lesions because the language areas are usually located in the left hemisphere. Speech problems caused by severe aphasia would compromise a consistent mode of delivery of communication between the CMR therapist and all participants. Thus, having left hemispheric lesions was one of the exclusion criteria in our study. Even though this homogeneous selection may limit the generalization of our findings, this selection enabled us to make the brain imaging data more easily interpretable.

## Results

### Demographics and behavioural findings

**Figure 2:**
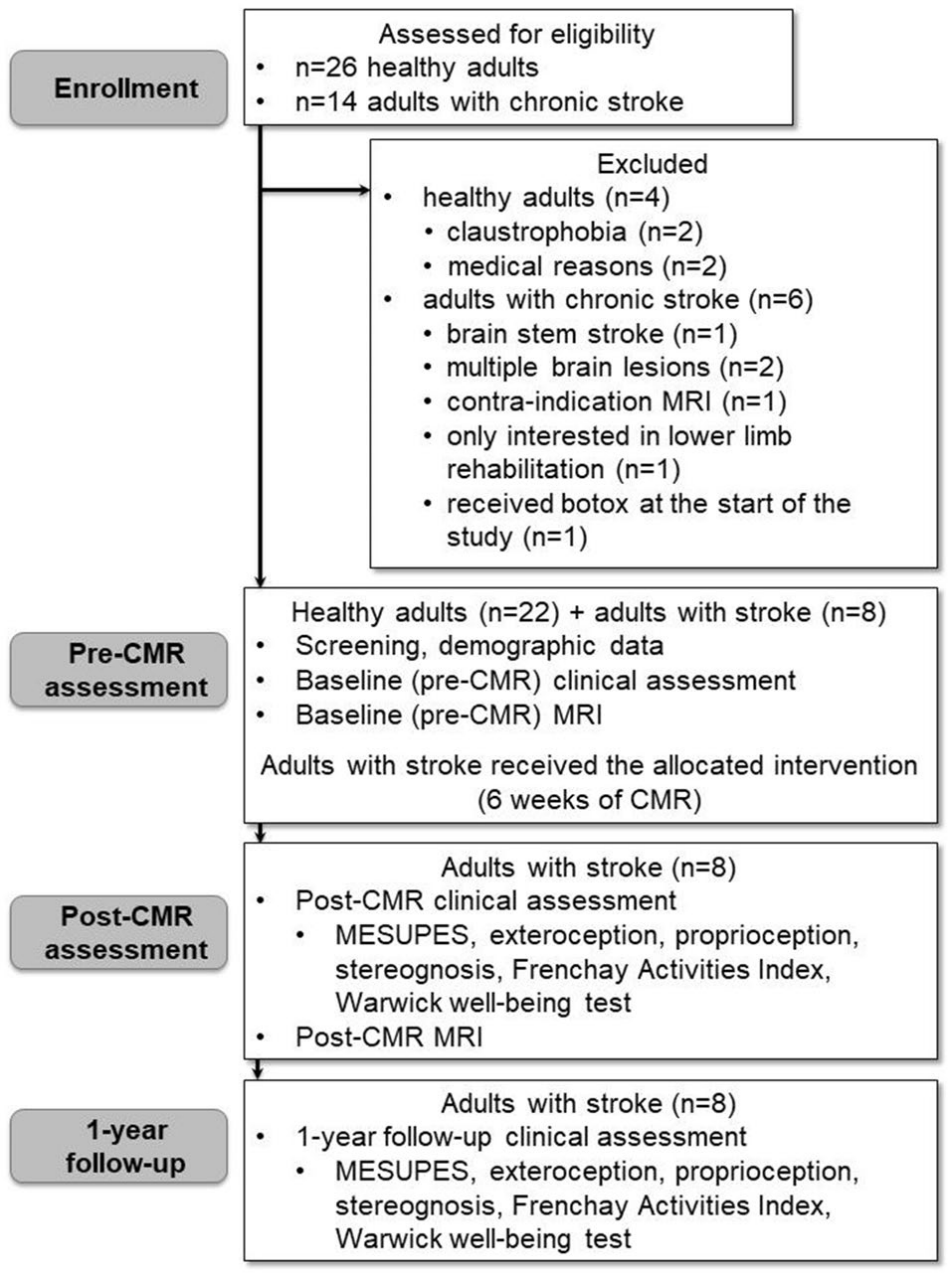
Flow chart. CMR: Cognitive Multisensory Rehabilitation; MESUPES: Motor Evaluation Scale for Upper Extremity in Stroke Patients; MRI: Magnetic Resonance Imaging.

Twenty-six healthy participants were contacted through convenience sampling. Of those, two had claustrophobia, and two did not participate for medical reasons. Of the 14 screened participants with chronic stroke, 6 were excluded: 5 because of multiple brain lesions or location outside of the middle cerebral artery (MCA) region; and 1 because this participant received botox at the beginning of the study. Figure 2 displays the flow chart. In sum, 22 healthy adults (median age [interquartile range] 64.50 [26.75] years, range 30 – 84 years, 14 women) and eight participants with chronic stroke (53.00 [13.75] years, range 28 – 73 years, 3 women, time since stroke (3.93 [5.56] years, range 1 – 7 years), all with an ischemic stroke lesion in the right MCA area with resulting left hemiplegia/hemiparesis participated in the study. The demographic and clinical data of both the healthy participants and participants with stroke are presented in Table 1. Healthy adults were tested at baseline only.

**Table 1:** Demographic and screening data of healthy participants and adults with stroke.

Median [IQR] for all outcome measures	Healthy adults (n = 22)	Adults with stroke (n = 8)	Statistics
**General baseline demographic assessments**			
Age (years, range)	64.50 [26.75] (30–84)	53.00 [13.75] (28–73)	*Z* = *−* *1.08, U* = *64.50* *p* = 0.28
Sex (females/males)	14/8	3/5	*Fisher’s Exact test p* = 0.24
Race (white/other)	21/1	7/1	*Fisher’s Exact test p* = 0.47
Ethnicity (not hispanic/hispanic)	22/0	8/0	*Fisher’s Exact test* *p* = 1.00
Edinburgh Handedness (left/mixed/right)	2/3/17	0/0/8	
Education (years, range)	17.75 [2.75] (14–21.5)	15.00 [2.50] (12–17)	*Z* = *−* *3.16, U* = *21.00* p = 0.002*
Corsi block span forwards (5 or 6 is a normal score)	6.00 [2.00] (5–9)	6.00 [1.75] (5–9)	*Z* = *−* *0.08, U* = *82.00* *p* = 0.94
Corsi block span backwards	7.00 [2.00] (4–9)	5.50 [3.00] (4–8)	*Z* = *−* *1.25, U* = *58.50* *p* = 0.21
Raven (percentage, range)	71.00 [31.00] (0–99)	35.00 [41.75] (14–75)	*Z* = *−* *2.39, U* = *34.50, *p = 0.002*
**Stroke-specific scales for screening and baseline assessment**			
Bell’s test (total scores 35)	34.00 [2.75]	35.00 [1.00]	*Z* = *1.26, U* = *114, p* = 0.21
MMSE-2:BV (total score 16, range)	16.00 [1.00] (14–16)	15.00 [1.00] (14–16)	*Z* = *−* *0.94, U* = *69.50,* *p* = 0.34
Apraxia test (total score 12)	12.00 [0.75]	10.50 [1.25]	*Z* = *−* *2.38, U* = *43.00* p = 0.02*
Aphasia rapid test (0 is a normal score)	0.00 [0.00]	0.00 [0.25]	*Z* = *1.15, U* = *103.00* *p* = 0.25
Numeric pain rating scale (range 0–10; 0 = no pain)	0.00 [1.00]	0.50 [2.00]	*Z* = *0.48, U* = *93.50* *p* = 0.63
MESUPES arm L (total 40)	40.00 [0.00]	25.00 [21.00]	*Z* = *−* *4.84, U* = *11.00* p < 0.0001*
MESUPES hand L (total 18)	18.00 [1.00]	3.00 [9.00]	*Z* = *−* *3.73, U* = *14.50* p = 0.0002*
MESUPES total L (total 58)	58.00 [1.00]	30.00 [27.00]	*Z* = *−* *3.73, U* = *14.50* p = 0.0002*
Exteroception index, thumb, palm (total 6)	6.00 [0.00]	5.00 [3.25]	*Z* = *−* *3.45, U* = *44.00* p = 0.0006*
Proprioception wrist, index (total 6)	8.00 [0.00]	8.00 [0.25]	*Z* = *−* *2.33, U* = *66.00* p = 0.02*
Stereognosis (total 6 objects)	6.00 [0.00]	2.50 [3.25]	*Z* = *−* *4.84, U* = *11.00* p < 0.0001*

* = statistically ssignificant, *p* < 0.05; All analyses are two-tailed and set at α = 0.05; IQR = interquartile range; MMSE-2:BV = Mini-Mental State Examination, 2nd Edition-Brief Version; Mann–Whitney U test: With samples of n > 20, the value of U approaches a normal distribution, and so the null hypothesis can be tested with a *Z*-test; MESUPES = Motor Evaluation Scale for Upper Extremity in Stroke Patients.

Regarding general baseline data (Table 1), only education (*p* = 0.002) and the Raven test (*p* = 0.02) were significantly lower in adults with stroke compared to healthy adults; other comparisons were not significantly different between both groups. As expected, stroke-specific scales such as motor function [Motor Evaluation for Upper Extremity in Stroke Patients (MESUPES) arm function *p* < 0.0001; hand function *p* = 0.0002, total score *p* = 0.0002], apraxia (*p* = 0.02), exteroception (*p* = 0.0006), proprioception (*p* = 0.02), and stereognosis (*p* < 0.0001) were significantly impaired in stroke at baseline compared to healthy adults.

The clinical data in participants with stroke at 3 time points (pre-CMR, post-CMR, and at 1-year follow-up) with statistical results from the Friedman test are presented in the Supplementary Table S1.

The Friedman test revealed significant changes over time for the MESUPES arm motor function (*p* = 0.02), for hand motor function (*p* = 0.03) and for the total score for upper limb motor function (*p* = 0.006). The Conover post-hoc tests with false discovery rate (FDR)-adjusted *p*-value showed that the three outcome measures of motor function improved between pre- and post-CMR (respectively, *p* = 0.0004; *p* = 0.0007; *p* = 0.0008) and between pre-CMR and 1-year follow-up (respectively, *p* = 0.001; *p* = 0.002; *p* = 0.0008).

The Friedman test also revealed significant changes over time for stereognosis (*p* = 0.03) and for the Frenchay Activities Index (*p* = 0.03). Those outcome measures improved from pre- to post-CMR (respectively, *p* = 0.004; *p* = 0.01) and from pre-CMR to 1-year follow-up (respectively, *p* = 0.001; *p* = 0.0008), reflecting improvements in sensory function and in participation in daily activities respectively. Of note is that the sensorimotor benefits were found after all participants with stroke had completed their rehabilitation care in the clinic and as outpatients.

**Figure 3:**
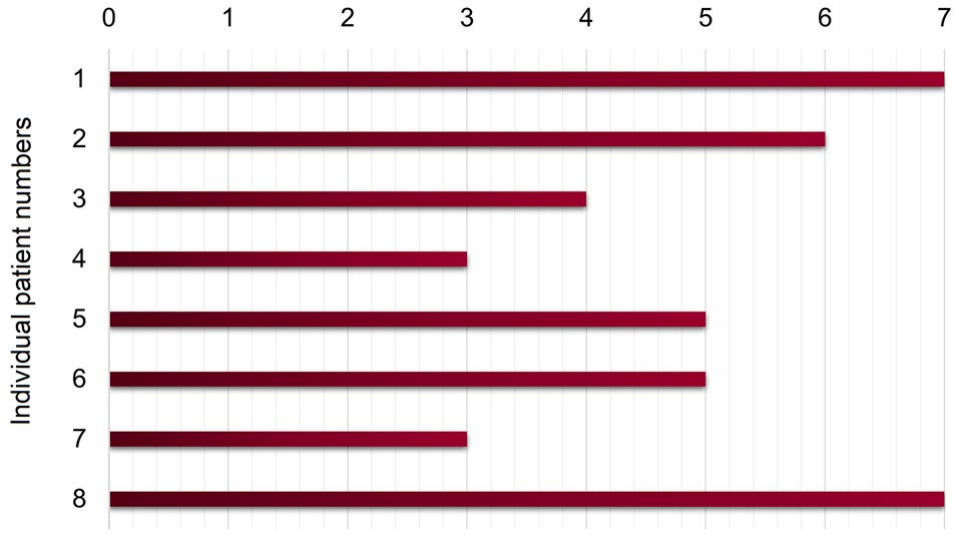
Global Rating of Change (GROC) scale. The scale ranges from − 7 (a very great deal worse) to 7 (a very great deal better). The depicted range of the 8 patients at 1-year follow-up are depicted below: score 2: a little bit better; score 3: somewhat better; score 4: moderately better; score 5: quite a bit better; score 6: a great deal better; score 7: a very great deal better.

GROC at 1-year follow-up are displayed per patient in Fig. 3. The participants with stroke graded their upper limb recovery at 1-year follow-up and reported GROC scores between 2 (i.e., a little bit better) and 7 (i.e., a very great deal better).

### Resting-state connectivity in healthy adults and adults with stroke

**Figure 4:**
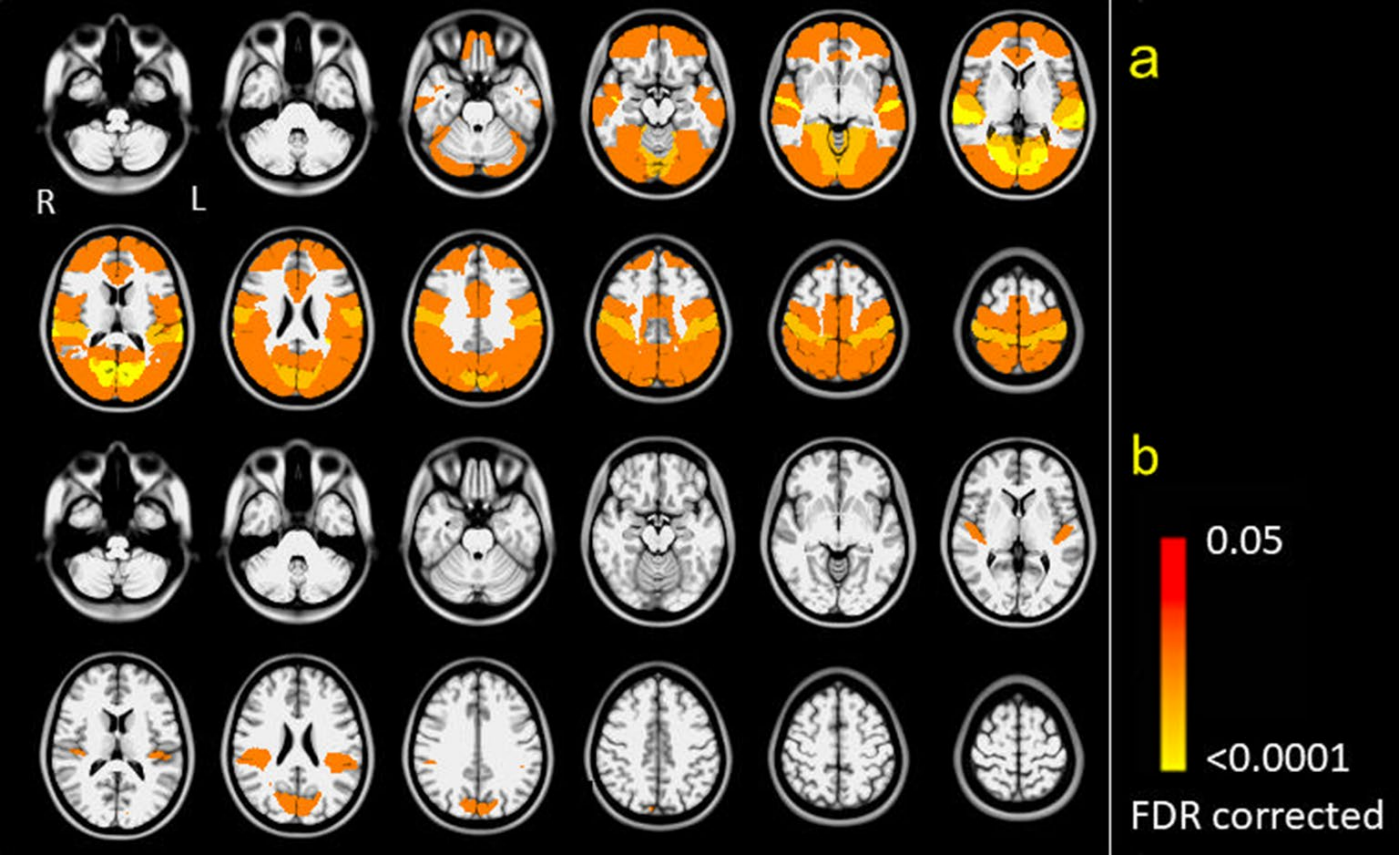
Comparison of functional connectivity between adults with stroke and healthy participants before (**a**) and after CMR (**b**). The yellow to red coloured regions from the Harvard–Oxford cortical atlas show areas whose functional connectivity with the OP1/OP4 seed region is statistically significantly stronger (FDR-adjusted *p*-values < 0.05) in healthy controls versus stroke pre-CMR (**a**) and in healthy controls versus stroke post-CMR (**b**).

At baseline, the resting-state functional analysis showed decreased FC between the right OP1/OP4 (lesioned hemisphere) and 34 areas distributed across all lobes in adults with stroke compared to healthy adults. After CMR, only four of those areas were significantly reduced in stroke compared to healthy adults: the right temporal pole, left cuneal cortex, left parietal operculum and left Heschl’s gyrus (Supplementary Table S2). Figure 4 depicts a comparison between stroke and healthy adults in terms of connectivity before and after CMR. The regions from the Harvard–Oxford cortical atlas were used to demonstrate statistically significantly greater functional connectivity with the OP1/OP4 seed region (FDR-adjusted *p*-values < 0.05) in healthy controls compared to the stroke group pre-CMR (a) and in healthy controls versus the stroke group post-CMR (b).

**Figure 5:**
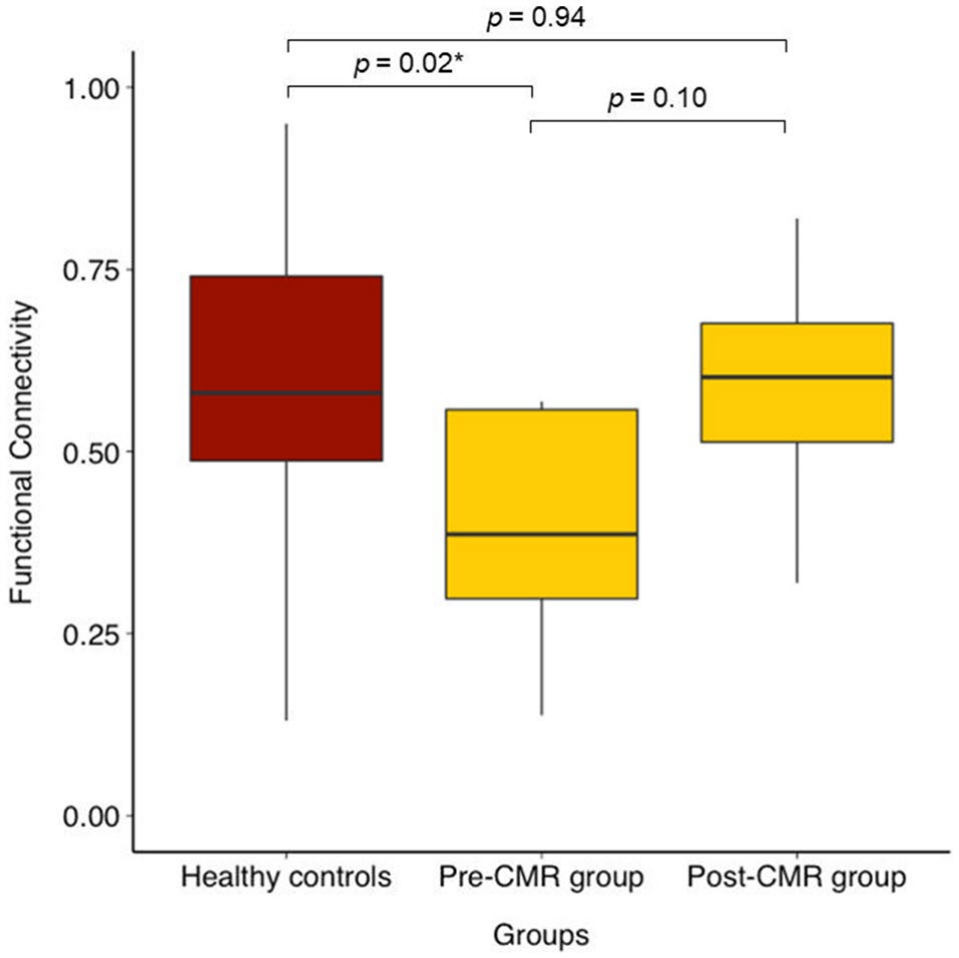
Boxplots of connectivity between the right parietal operculum and the right superior parietal lobe. The boxplots illustrate an example of functional connectivity in healthy controls compared to adults with stroke, pre-CMR, and post-CMR, between the OP1/OP4 and the right superior parietal lobe. The star depicts a statistically significant FDR-adjusted *p*-value showing weaker connectivity strength in the stroke group at baseline (pre-CMR) compared to the healthy controls. Post-CMR, there was no statistically significant difference in connectivity strength between the stroke group and the healthy controls.

Further, as an illustration, Fig. 5 depicts boxplots for the healthy controls, and for pre-CMR, post-CMR in stroke. The boxplots show significantly decreased FC between the right OP1/OP4 and the right superior parietal lobe pre-CMR when adults with stroke were compared to healthy controls. However, after CMR, there was no statistically significant difference in connectivity strength between adults with stroke and healthy controls.

## Discussion

Our behavioural results regarding motor function and participation in daily activities are in alignment with the previously reported CMR studies in adults with acute and chronic stroke^15,16^, even though different outcome measures were used to evaluate motor recovery and participation of daily activities than those reported in the two previous studies. Nevertheless, our overall results point to the validity of our pilot clinical study regarding the effect of CMR on behavioural outcomes.

To the best of our knowledge, this is the first brain imaging study, which explored brain connectivity changes related to CMR. These exploratory results support the concept that, after a stroke, neurorehabilitation can target neuroplasticity by increasing connectivity in sensory and motor networks^27,28^. Our results expand further knowledge on recovery of sensorimotor networks following rehabilitation.

**Figure 6:**
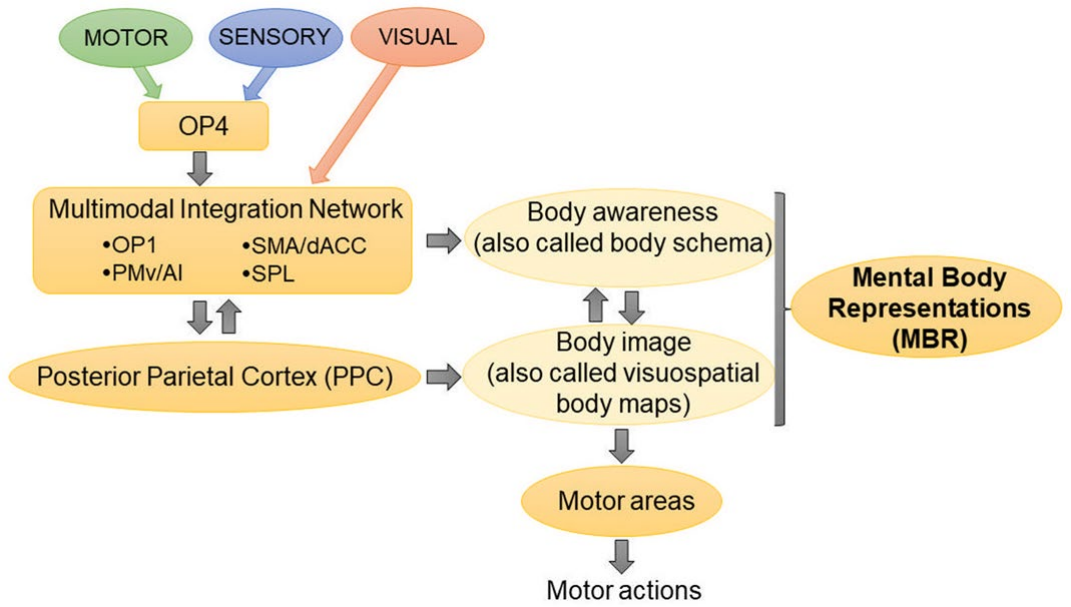
Proposed model to situate mental body representations within the currently known multi-sensorimotor cycle in the brain, which ultimately leads to the generation of motor actions. AG, angular gyrus; AI, anterior insula; dACC, dorsal anterior cingulate cortex; OP1/OP4, parietal operculum parts 1 and 4; PMv, ventral premotor cortex; SMA, supplementary motor area; SMG, supramarginal gyrus; SPL, superior parietal lobe.

In this study, we observed that, after CMR, many of the 30 restored connections were connections between OP1/OP4 and other areas within the multisensory integration network (Fig. 6). This network provides essential information in order to generate and perform accurate motor actions^23,24,29–32^. Therefore, it is likely that restoration of connections within this network that includes OP1/OP4 played a role in the recovery of motor function. Furthermore, the importance of OP1/OP4 for their potential role in motor recovery is strengthened by the finding that OP1/OP4 activity measured during acute stroke appears to be a good indicator of how well motor function could be recovered 6 months later in adults with large hemispheric strokes and moderate to severe motor impairments^33^. How exactly activation of OP1/OP4 leads to restoration of connectivity in the brain relevant for sensorimotor function remains unknown and will be the subject of our future investigations.

The underlying mechanism of how the cognitive processing, reflection, and attention to sensorimotor tasks during CMR influences sensorimotor recovery also remains largely unknown and is the main subject of our ongoing investigation. Nonetheless, the current understanding of how the integration of multisensory information influences motor function at the brain level, as illustrated in Fig. 6, may provide a clue as to how CMR may function to restore motor function.

In CMR exercises, the therapist will probe the patient through questions to consciously reflect on the position of their arm and hand in space and to have a focused awareness to the multisensory processing and their movements during sensory discrimination exercises. In the end, the CMR exercises probably help adults with stroke recalibrate what is called their “mental body representations” (MBR). MBR refers to accurate updated information about body positions and movements in space at any moment, and thus MBR is critical to performing motor actions correctly^34,35^. Additionally, recent literature has shown that 81% of adults with vascular brain injuries exhibit deficits in at least one type of MBR^36^.

How MBR may fit into the current understanding of the multi-sensorimotor cycle in the brain is illustrated in Fig. 6. Our proposed model in Fig. 6 is constructed based on the multimodal integration network model derived from resting-state functional connectivity data in healthy adults^23,24^; on our previous work^17–20^; and on the work of others^21,30,37–40^. In this model, information from the primary sensory and motor cortex reaches the multimodal integration network via OP4. Within this network sensory, motor, and visual information is converged^23,24^ to form body awareness, also called body schema^21,30,37–40^. Body awareness in this context, refers to a perceptual understanding and awareness of (i) proprioception: body position and movement sense and how those body parts are situated in peripersonal space, (ii) exteroception: visual, tactile, auditory signals locations within the peripersonal space; (iii) interoception: internal body states^21,30,37–40^. Then, the information formed in the multimodal integration network is sent to the posterior PPC in which the body image is formed^35,41^. Body image refers to dynamic visuospatial body maps that house a cognitive understanding and awareness of how the whole body is situated in space at each timepoint^35,36,41,42^. Body awareness and body image are two different types of MBR and they interact with each other^43,44^. Finally, the body image is necessary to guide execution of precise motor actions, such as hand movements (Fig. 6)^34^.

Considering this model, CMR exercises are targeting the restoration of body awareness directly, which in turn, directly and indirectly improves body image and thus MBR as a whole, which aids restoration of motor function. At the brain function level, we speculate that (i) OP1/OP4 in the multimodal integration network plays a crucial role in the formation of accurate body awareness, and that (ii) improvement of body awareness by CMR may activate OP1/OP4, leading to restoration of the brain connectivity that we have observed.

As stated in the introduction, we only reported on results in adults with left hemiplegia or hemiparesis, which may limit the generalization of possible effects for adults with right hemiplegia or hemiparesis. Nonetheless, we have demonstrated in our prior fMRI studies^19^ that CMR-derived exercises in the scanner produce the same frontoparietal activation in adults with right hemiplegia/hemiparesis than we found in adults with left hemiplegia/hemiparesis who were scanned in our more recent studies. Thus, it is possible that similar brain changes might be found after CMR for adults with right hemiplegia or hemiparesis. The presented data justify future studies to investigate changes in brain activation and connections after CMR in a larger sample of adults with stroke.

## Methods

### Participants

All participants were recruited between November 2015 and August 2017. The participants signed an informed consent, and the study was performed in accordance with the Declaration of Helsinki. The Institutional Review Board (IRB) of the University of Minnesota approved the study.

Adults with stroke were included if they (i) were between 18–85 years old, (ii) were at least 6 months after a stroke that occurred in adulthood, (ii) had a stable ischemic infarct with a lesion in the right MCA region, with resulting left hemiplegia/paresis, (iv) did not receive ongoing rehabilitation at the time of testing, and (v) were able to read, hear and comprehend instructions in English. Exclusion criteria were: (i) contra-indications for MRI scanning; (ii) other medical conditions precluding full participation, including (other) brain injuries/illnesses, cognitive impairment, severe unilateral spatial neglect or severe proprioceptive loss, hindering them to feel finger movements on the affected side, severe apraxia, severe aphasia, or contractures that restricted them from keeping the outstretched arm in a relaxed position.

Healthy adults of similar age than adults with stroke were recruited through fliers and through the University website. Healthy participants had to be medically stable; and able to read, hear and comprehend instructions in English. We excluded healthy adults who had brain injuries, who had complete proprioceptive loss, contractures in the arm, who were medically unstable, had cognitive impairments, or for whom MRI scanning was contra-indicated.

### Research design

This was an exploratory pilot study with a single arm pre-post experimental design in which participants with stroke were tested with behavioural tests, structural and resting-state functional MRI at baseline; behavioural tests, structural and resting-state functional MRI after 6 weeks of therapy (CMR, 3 times a week for 45 min/session), and behavioural tests at follow-up, 1 year after the therapy had ended. Healthy volunteers were recruited as a control group and were tested once on behavioural tests, structural and resting-state functional MRI. The evaluator who performed the screening, behavioural tests, and MRI assessment was a different person than the therapists giving the intervention.

### Clinical assessments

All participants (i.e., healthy adults as well as adults with stroke) underwent screening tests, i.e., Bell’s test for neglect (cut-off < 29/35)^45^; Mini-Mental State Examination, 2nd Edition-Brief Version (MMSE-2:BV, cut-off < 13/16)^46^; Apraxia Screen test (cut-off < 5/12 for severe apraxia)^47^; Aphasia Rapid Test [cut-off > 21/26 (lower score is better outcome)]^48^; Edinburgh Handedness Inventory^49^; Raven’s Progressive Matrices to measure abstract reasoning^50^; the Corsi Block Tapping Task^51^ to test visuo-spatial short-term memory; exteroceptive sensibility (tactile touch); proprioceptive sensibility (position and motion sense); stereognosis, during which participants need to correctly identify objects by feeling the object in the hand with eyes closed^52^; and the Numeric Pain Rating scale^53^ to identify pain in upper limb. Participants also completed medical and general health questionnaires.

Motor function of the affected arm in adults with stroke was evaluated with the MESUPES for which the therapists assigned scores on the perceived muscle tone during passive arm movements; on assisted and active arm movements, and on active hand and finger movements, including dexterity tasks (e.g., rotating a dice with thumb and index finger)^5,54^. Secondary clinical outcome measures in adults with stroke were the exteroceptive and proprioceptive sensibility^52^; stereognosis^52^; the Frenchay Activities Index^55^, which enquires how often participants are engaged in daily activities; and the Warwick-Edinburgh Mental Well-Being Scale^56^. At 1-year follow-up, we asked the participants to grade their perceived level of stroke recovery in terms of upper limb motor function after therapy and follow-up period, ranging from − 7 (i.e., a very great deal worse) to 7 (i.e., a very great deal better)^57^.

### Cognitive multisensory rehabilitation (CMR)

CMR is also translated in other publications as cognitive therapeutic exercises^58^, Cognitive Sensory Motor Training^16^, neurocognitive therapeutic exercise^59^, cognitive exercise therapy^15^, Perfetti method^16^, or (neuro)cognitive approach^60,61^.

CMR incorporates *conscious* perception of body positions and movements during (multi)sensory discrimination exercises^12–14^. The treating therapists, with years of clinical practice and specialized in this therapy, gave 35 min of discrimination exercises embedded in functional movements followed by 10 min of applying the learned strategies during activities of daily living. The difficulty of the exercises was set at ± 75% success, monitored by the number of correct responses given to an exercise, to promote learning, engage motivation and avoid frustration.

CMR uses several types of discrimination exercises: Participants discriminated shapes, length, weight, distance, resistance, textures or compared kinaesthetic information with visual information for integration of multisensory information^13^. Two examples are given in Fig. 1. The election of the exercises is chosen based on what the participant experiences difficulty with. For example, the participant might experience hypertonia in a certain range of motion of the shoulder for which the exercise in Fig. 1a might be useful. If the participant has sensory loss in the fingers, the therapist might opt to use texture discrimination exercises. Solving the discrimination task is combined with reflection and a learning process, prompted by the therapist on how the limb (was) moved or was positioned or, in case of sensory loss, how the sensation was perceived on the finger pads during the texture discrimination. The discrimination exercise itself is done with eyes closed to avoid compensating with vision, but afterwards the improved proprioceptive ability is integrated during functional tasks while participants have their eyes open. Assistance to the movements is given as much as needed to perform a correct motion and is gradually reduced to encourage independent function.

Focusing attention on movements helped participants control their muscle tone and relax their hand^62,63^, thereby creating the potential for movements to re-emerge. Exercises focused on activating only those muscles relevant for the movement, adapting a correct relaxed sensation of the movement in proper form; relate and compare movements with the affected and unaffected arm; understanding the different movement components and how they relate to each other; and recognizing spatial and temporal cues.

Furthermore, kinaesthetic motor imagery was included to increase correct sense perception and, if present, to decrease pain^14,59^. Depending on the severity of the upper limb motor impairment, exercises transitioned from passive, assisted, active, to functional movements progressively throughout the therapy, based on the level of motor function ability of the participant to correctly perform the movement. All activities were directly related to functional tasks. Several body joints were included in the exercise to integrate speed and dexterity in functional movements.

### Structural and resting-state functional MRI (fMRI) acquisition and pre-processing

Structural MRI acquisition was acquired using a T1 weighted MPRAGE image [TR = 2.5 s, TE = 3.65 ms, 1 mm^3^ voxels], as well as T2 weighted SPACE image [TR = 3 s, TE = 565 ms, 1 mm^3^ voxels], and SPACE based FLAIR [TR = 5.0 s, TE = 394 ms, 1 mm^3^ voxels] images to quantify lesion extent. The 3 T Siemens auto-align longitudinal repositioning system was used to ensure comparable head positioning across the scan sessions. Structural Preprocessing was performed using the containerized FMRIPREP^64^ version 1.2.2 with standardized BIDS formatted NIFTI data^65^. More information the details of this process can be found on the FMRIPREP website https://fmriprep.org/en/20.2.0/citing.html#. ICA-based Automatic Removal Of Motion Artifacts (AROMA) were used to generate aggressive noise regressors and to create a variant of data that was non-aggressively denoised^66^.

### Resting-state fMRI: set-up and statistical analysis

Participants underwent a resting-state fMRI scan of 14.50 min with eyes open, maintaining fixation with a restful mind and they were asked to not fall asleep. The Harvard–Oxford cortical atlas was split into regions corresponding to each of the left and right hemisphere, resulting in a total of 96 regions of interest (ROI). The resulting resting-state BOLD-contrast time series were extracted *from each ROI* from each participant by averaging the voxel-level time courses within each ROI. For each participant, Pearson correlation coefficients were computed to construct functional connectivity metrics between the OP1/OP4 in the affected (i.e., right) hemisphere, thereby serving as the seed region, in the correlation analysis with the other ROIs. Two-sample *t*-tests were used to determine differences between the participants with stroke and healthy controls in the FC between the seed region and each ROI. The Benjamini–Hochberg procedure for controlling the FDR was used to adjust the *p*-values to account for multiple comparisons.

### Behavioural data: statistical analysis

Behavioural data were calculated with JMP, Version 13, SAS Institute Inc., Cary, NC, 1989–2007. Nominal data were calculated with the Fisher Exact test between the healthy group and the stroke group at baseline (https://www.socscistatistics.com/tests/fisher/default2.aspx). Because of the small sample size, we used non-parametric statistics (Mann–Whitney U tests) for all comparisons of baseline data between the healthy group and the stroke group for outcome measures with ordinal or ratio levels of measurement. We used the Friedman test with post-hoc Conover *p*-values, further adjusted by the Benjamini–Hochberg FDR method to compare the clinical data in adults with stroke at baseline, after CMR, and at 1-year follow-up (https://astatsa.com/FriedmanTest/). The a-priori chosen level of significance was set at α = 0.05 and all statistical tests were two-tailed. Subgroup analyses for sex, race or ethnicity were not feasible due to the small sample size.

## Supplementary information

Supplementary Table S1.

Supplementary Table S2.

## Data Availability

The datasets generated and analysed during the current study are available from the corresponding author on reasonable request.
