# Supplementary material for: Exploratory study of how Cognitive Multisensory Rehabilitation restores parietal operculum connectivity and improves upper limb movements in chronic stroke
Source: Sci Rep. 2020 Nov 20;10:20278. doi: 10.1038/s41598-020-77272-y (PMC7680110; doi:10.1038/s41598-020-77272-y)
Supplement: Supplementary file 1 — Supplementary Table S1. [file 41598_2020_77272_MOESM1_ESM.docx]

**Supplementary Table S1.** Clinical data of participants with stroke at baseline, post-stroke and 1-year follow-up

|  | **Adults with stroke (n=8)** | | | |
| --- | --- | --- | --- | --- |
| **Clinical tests: Median [IQR]** | **Baseline (Pre-CMR)** | **Post-CMR** | **1-year Follow-up** | **Friedman test: *Χ^2^_r_* (DF), *p*-value** |
| MESUPES arm L (total 40) | 25.00 [21.00] | 28.00 [16.00] | 28.50 [8.75] | *Χ^2^_r_ (2)*=8.09, ***p*=0.02*** |
| MESUPES hand L (total 18) | 3.00 [9.00] | 5.50 [10.75] | 7.00 [10.75] | *Χ^2^_r_ (2)*=8.09, ***p*=0.03*** |
| MESUPES total L (total 58) | 30.00 [27.00] | 36.50 [23.25] | 35.50 [29.75] | *Χ^2^_r_ (2)*=10.16, ***p*=0.006*** |
| Exteroception: index finger, thumb, palm (total 6) | 5.00 [3.25] | 6.00 [1.00] | 6.00 [0.50] | *Χ^2^_r_ (2)*=1.31, *p*=0.52 |
| Proprioception: wrist, index finger (total 8) | 8.00 [0.25] | 8.00 [0.00] | 8.00 [0.00] | *Χ^2^_r_ (2)*=0.81, *p*=0.66 |
| Stereognosis (total 6 objects) | 2.50 [3.25] | 4.00 [1.50] | 5.50 [2.25] | *Χ^2^_r_ (2)*=6.87, ***p*=0.03*** |
| Frenchay Activities Index  (total 45) | 22.00 [12.00] | 25.50 [12.00] | 30.00 [14.75] | *Χ^2^_r_ (2)*=6.87, ***p*=0.03*** |
| Warwick Well-being scale  (total 70) | 53.50 [19.25] | 54.50 [11.25] | 60.50 [13.00] | *Χ^2^_r_ (2)*=2.44, *p*=0.30 |

**Legend:** ***** =statistically significant; All analyses are two-tailed and set at α=0.05; DF = degrees of freedom; *Χ^2^_r_* = Friedman chi-squared statistic; IQR = interquartile range; L = left; MESUPES = Motor Evaluation Scale for Upper Extremity in Stroke Patients
