# Supplementary material for: Exploratory study of how Cognitive Multisensory Rehabilitation restores parietal operculum connectivity and improves upper limb movements in chronic stroke
Source: Sci Rep. 2020 Nov 20;10:20278. doi: 10.1038/s41598-020-77272-y (PMC7680110; doi:10.1038/s41598-020-77272-y)
Supplement: Supplementary file 2 — Supplementary Table S2. [file 41598_2020_77272_MOESM2_ESM.docx]

**Supplementary Table S2.** Brain areas with significantly decreased connectivity with the right OP1/OP4 (lesioned hemisphere) in stroke compared to healthy adults.

| **Brain area (hemisphere)** | **Stroke ROI** | **Stroke < healthy** |  | **Stroke < healthy** |
| --- | --- | --- | --- | --- |
| **OP1/OP4 (R) connectivity with:** | **Right side** |  | **Left side** |  |
| ROI | ***t-statistic*** | ***Adjusted p-value*** | ***t-statistic*** | ***Adjusted  p-value*** |
| **PRE-CMR** | | | | |
| **Frontal lobe** | | | | |
| Frontal pole (RL) | 2.82 | 0.020 | 2.79 | 0.019 |
| Superior frontal gyrus (R) | 3.35 | 0.009 |  |  |
| Middle frontal gyrus (R) | 2.71 | 0.025 |  |  |
| Precentral gyrus (RL) | 3.33 | 0.007 | 3.66 | 0.003 |
| Frontal operculum cortex (R) | 3.04 | 0.010 |  |  |
| Supplementary motor area (RL) | 3.48 | 0.004 | 3.11 | 0.009 |
| Cingulate gyrus, anterior division (RL) | 2.73 | 0.022 | 2.98 | 0.013 |
| Paracingulate gyrus (R) | 2.65 | 0.025 |  |  |
| **Temporal lobe** | | | | |
| Superior temporal gyrus, anterior division (RL) | 3.61 | 0.004 | 3.38 | 0.003 |
| Superior temporal gyrus, posterior division (RL) | 3.93 | 0.002 | 5.01 | <0.0001 |
| Middle temporal gyrus, posterior division (RL) | 3.35 | 0.009 | 2.75 | 0.017 |
| Planum polare (RL) | 3.30 | 0.008 | 2.51 | 0.028 |
| Planum temporale (RL) | 4.38 | 0.001 | 4.15 | 0.002 |
| Heschl’s gyrus (RL) | 3.04 | 0.010 | 4.07 | 0.001 |
| Temporal pole (R) | 3.45 | 0.005 |  |  |
| **Parietal lobe** | | | | |
| Postcentral gyrus (RL) | 3.08 | 0.013 | 3.91 | 0.002 |
| Superior parietal lobe (RL) | 2.56 | 0.021 | 2.74 | 0.020 |
| Supramarginal gyrus, anterior division (L) |  |  | 3.53 | 0.004 |
| Supramarginal gyrus, posterior division (L) |  |  | 3.49 | 0.005 |
| Angular gyrus (L) |  |  | 2.78 | 0.017 |
| Parietal operculum cortex (L) |  |  | 3.27 | 0.010 |
| Frontoparietal operculum cortex (L) | 2.74 | 0.019 | 2.58 | 0.022 |
| Insular cortex (R) | 2.60 | 0.029 |  |  |
| Precuneus (RL) | 4.26 | 0.001 | 3.25 | 0.006 |
| Cingulate gyrus, posterior division (R) | 2.57 | 0.023 |  |  |
| **Occipital lobe** | | | | |
| Lateral occipital cortex, superior division (RL) | 3.63 | 0.005 | 3.38 | 0.004 |
| Lateral occipital cortex, inferior division (RL) | 3.33 | 0.006 | 2.99 | 0.007 |
| Intracalcarine cortex (RL) | 4.71 | <0.001 | 4.71 | <0.001 |
| Cuneal cortex (RL) | 3.40 | 0.004 | 4.10 | 0.001 |
| Lingual gyrus (RL) | 4.34 | <0.001 | 3.83 | 0.001 |
| Temporo-occipital fusiform gyrus (RL) | 2.58 | 0.021 | 2.39 | 0.030 |
| Occipital fusiform gyrus (RL) | 5.06 | <0.001 | 2.83 | 0.011 |
| Supracalcarine cortex (RL) | 4.75 | <0.001 | 4.01 | <0.001 |
| Occipital pole (RL) | 3.69 | 0.002 | 3.05 | 0.007 |
| **POST-CMR** | | | | |
| **Temporal lobe** | | | | |
| Temporal pole (R) | 4.18 | 0.001 |  |  |
| Heschl’s gyrus (L) |  |  | 4.48 | <0.001 |
| **Parietal lobe** | | | | |
| Parietal operculum cortex (L) |  |  | 4.32 | 0.001 |
| **Occipital lobe** | | | | |
| Cuneal cortex (L) |  |  | 3.99 | 0.001 |

**Legend:** The Benjamini-Hochberg procedure for controlling the false discovery rate (FDR) was used to adjust the *p*-values to account for multiple comparisons.
